# Supplementary material for: Energy conservation by collective movement in schooling fish
Source: eLife. 2024 Feb 20;12:RP90352. doi: 10.7554/eLife.90352 (PMC10942612; doi:10.7554/eLife.90352)
Supplement: Supplementary file 1. — These materials are a supplement to the main text and explain the aspects of the methods in more detail and document how we measured both the aerobic and non-aerobic contributions to total energy expenditure (TEE) during locomotion both by individuals and by the replicate schools of giant danio. Also included in this supplemental material are tables that summarize the various terms used for fish group locomotor behaviour and literature on fish schooling swim speeds in nature. We would like to emphasize that we focus on active, directional fish schooling behaviour and that such behaviour in the wild involves fish swimming at relatively high speeds, up to 20 body lengths second-1. This contrasts with many previous studies of fish schooling that have been conducted in still water. When fish swim at high speeds they engage in anaerobic glycolysis, and this can contribute significantly to the total cost of swimming. In this study, we measure both the aerobic and non-aerobic costs of active directional schooling behaviour in fishes, and by studying fish groups swimming at higher speeds we demonstrate directly the substantial energy savings resulting from group locomotion. [file elife-90352-supp1.docx]

Supplementary File for

**Energy conservation by collective movement in schooling fish**

Yangfan Zhang^1^*, George Lauder^1^

^1^Department of Organismic and Evolutionary Biology, Harvard University; Cambridge, Massachusetts, USA

*Corresponding author. Email: yangfan_zhang@fas.harvard.edu

**This file includes:**

Supplement File 1a–1d pp. 2 – 10

| **Supplement file 1a.** **A summary of the diversity of terms that have been used to describe fish collective behaviour**. Swim speeds measured in most studies are relatively low, and only a few measurements are available for active, directional schooling where high swimming speeds > 5 BL s^-1^ are used by fish groups (see Table S3). BL = body lengths. | | | |  |
| --- | --- | --- | --- | --- |
| **Descriptive term** | **Typical behaviour/use of term** | **Approximate individual swim speed range** | **Sample reference** | |
| Grouping | Non-specific aggregation of fishes, often used to describe fish foraging or moving about as an ecological strategy | 0 - 2 BL s^-1^ | Polyakov et al., *Science Advances*. **8**, (26) (2022) | |
| Aggregating | Individuals in the group in various orientations; social interactions among group members; low mean directional group motion; individuals frequently change position relative to conspecifics | 0 - 2 BL s^-1^ | Rountree, *Bulletin of Marine Science*. **4**, 960-972 (1989) | |
| Milling | Active swimming at lower speeds, most often in a circular motion in a torus configuration with a low net forward speed of the group as a whole | 0.25 - 3 BL s^-1^ | Costanzo and Hemelrijk, *Journal of Physics D: Applied Physics*. **51**, 134004 (2018) | |
| Shoaling | Individuals in the group in various orientations; low mean directional group motion; social interactions among group members; food searching; individuals frequently change position relative to conspecifics | 0.25 - 2 BL s^-1^ | E. Hensor et al., *Oikos*. **110**, 344-352 (2005) | |
| Migrating | Active long-distance directional swimming at speeds near the minimum cost of transport | 0.5 - 1.5 BL s^-1^ | Jorgensen et al. (2009). *Pro. Royal Soc. Lond. B,*  rspb20091155. | |
| Schooling: non-directional | Group swimming but with low mean group forward speed; predator avoidance bait ball conformation; individuals frequently change position relative to conspecifics | 1.0 - 4 BL s^-1^ | Mekdara et al., *Integrative and comparative biology*. **61**, 427-441 (2021) | |
| Schooling: active, directional | Group swimming with most individuals having similar body orientation and with directed higher speed forward motion of the group as a whole; frequency of individuals changing position within the group declines as speed increases | 0.25 - 20 BL s^-1^ | Anras et al., *Canadian Journal of Fisheries and Aquatic Sciences*. **54**, 162-168  (1997); This study. | |

**Supplement file 1b.** **Parameters for the metabolic model for calculating the total O_2_ cost during a critical swimming speed (*U*_crit_) test.** This model adds the amount of O_2_ consumed post-*U*_crit_ test (excess post-exercise O_2_ consumption, EPOC, calculated by an area under the curve algorithm, AUC) on the active O_2_ uptake (*Ṁ*O_2_) over the speed range of 4­­­–8 body lengths per second (BL s^-1^) when fish use ≥ 40% maximum *Ṁ*O_2_ (*Ṁ*O_2max_). A threshold level of 40% *Ṁ*O_2max_ is the workload that initiates glycolytic metabolism. This metabolic cost is a major contributor to EPOC. The model uses a percentage (%) modifier to compute the O_2_ cost in addition to the active *Ṁ*O_2_ at each swimming speed. The key criterion is that the area of non-aerobic O_2_ cost above the *Ṁ*O_2_ should be equal to EPOC, as shown in the section of the table for EPOC validation. The added area of non-aerobic O_2_ cost is calculated by the delta of AUC for the measured *Ṁ*O_2_ and the AUC for the *Ṁ*O_2_ model. As a result, the calculated total energy expenditure is modeled by the same equation for the theoretical relationship between total power and swimming speed (Fig. S8). Modeling is performed on each school and each solitary individual to calculate the 95% C.I. (*see* Fig. 2).

|  | **Model parameters** | |  |  |  | **EPOC validation** | |  |  | |  |  |
| --- | --- | --- | --- | --- | --- | --- | --- | --- | --- | --- | --- | --- |
|  | Swim speed (BL s^-1^) | *Ṁ*O_2_ (mg O_2_h^-1^kg^-1^) | Modeled *Ṁ*O_2_ (mg O_2_ h^-1^kg^-1^) | % Modifier | %  *Ṁ*O_2max_ | AUC of measure  (mg O_2_ kg^-1^) | AUC of model  (mg O_2_ kg^-1^) | Delta AUC (model-measure) (mg O_2_ kg^-1^) | | EPOC  (mg O_2_ kg^-1^) | % Difference of Delta AUC & EPOC | |
| School 1 | 4 | 455 | 550 | 21 | 40% | 1269 | 885.8 | 383 | | 383 | 0% | |
|  | 5 | 504 | 720 | 43 | 44% |  |  |  | |  |  | |
|  | 6 | 777 | 1204 | 55 | 68% |  |  |  | |  |  | |
|  | 7 | 930 | 1777 | 91 | 81% |  |  |  | |  |  | |
|  | 8 | 1146 | 2567 | 124 | 100% |  |  |  | |  |  | |
| School 2 | 4 | 508 | 625 | 23 | 46% | 1359 | 944 | 415 | | 414 | 0% | |
|  | 5 | 694 | 979 | 41 | 63% |  |  |  | |  |  | |
|  | 6 | 769 | 1277 | 66 | 70% |  |  |  | |  |  | |
|  | 7 | 950 | 1825 | 92 | 86% |  |  |  | |  |  | |
|  | 8 | 1105 | 2520 | 128 | 100% |  |  |  | |  |  | |
| School 3 | 4 | 565 | 706 | 25 | 59% | 1340 | 920.5 | 420 | | 420 | 0% | |
|  | 5 | 544 | 779 | 43 | 56% |  |  |  | |  |  | |
|  | 6 | 670 | 1179 | 76 | 69% |  |  |  | |  |  | |
|  | 7 | 724 | 1636 | 126 | 75% |  |  |  | |  |  | |
|  | 8 | 965 | 2413 | 150 | 100% |  |  |  | |  |  | |
| School 4 | 4 | 484 | 639 | 32 | 47% | 1539 | 941.4 | 598 | | 598 | 0% | |
|  | 5 | 639 | 1003 | 57 | 61% |  |  |  | |  |  | |
|  | 6 | 687 | 1360 | 98 | 66% |  |  |  | |  |  | |
|  | 7 | 858 | 2162 | 152 | 83% |  |  |  | |  |  | |
|  | 8 | 1039 | 3222 | 210 | 100% |  |  |  | |  |  | |
| School 5 | 4 | 454 | 508 | 12 | 46% | 1074 | 821 | 253 | | 253 | 0% | |
|  | 5 | 511 | 644 | 26 | 51% |  |  |  | |  |  | |
|  | 6 | 637 | 860 | 35 | 64% |  |  |  | |  |  | |
|  | 7 | 740 | 1302 | 76 | 74% |  |  |  | |  |  | |
|  | 8 | 994 | 2088 | 110 | 100% |  |  |  | |  |  | |
| Individual 1 | 4 | 473 | 756 | 60 | 65% | 766.6 | 1504 | 737 | | 736 | 0% | |
|  | 5 | 549 | 1087 | 98 | 76% |  |  |  | |  |  | |
|  | 6 | 700 | 1680 | 140 | 96% |  |  |  | |  |  | |
|  | 7 | 722 | 2312 | 220 | 99% |  |  |  | |  |  | |
|  | 8 | 726 | 2789 | 284 | 100% |  |  |  | |  |  | |
| Individual 2 | 4 | 563 | 1070 | 90 | 66% | 705.7 | 2224 | 1518 | | 1517 | -0.09% | |
|  | 5 | 531 | 1592 | 200 | 62% |  |  |  | |  |  | |
|  | 6 | 562 | 2351 | 318 | 66% |  |  |  | |  |  | |
|  | 7 | 849 | 4331 | 410 | 100% |  |  |  | |  |  | |
|  | 8 | 808 | 5347 | 562 | 95% |  |  |  | |  |  | |
| Individual 3 | 4 | 447 | 671 | 50 | 50% | 874.6 | 2138 | 1263 | | 1264 | 0.05% | |
|  | 5 | 593 | 1481 | 150 | 66% |  |  |  | |  |  | |
|  | 6 | 712 | 2564 | 260 | 79% |  |  |  | |  |  | |
|  | 7 | 903 | 3701 | 310 | 100% |  |  |  | |  |  | |
|  | 8 | 668 | 4307 | 545 | 74% |  |  |  | |  |  | |
| Individual 4 | 4 | 334 | 834 | 150 | 50% | 747.1 | 1618 | 871 | | 870 | -0.09% | |
|  | 5 | 336 | 1243 | 270 | 50% |  |  |  | |  |  | |
|  | 6 | 596 | 2531 | 325 | 89% |  |  |  | |  |  | |
|  | 7 | 671 | 4431 | 560 | 100% |  |  |  | |  |  | |

| **Supplement file 1c.** **A summary table of schooling speeds and seasonal migratory speeds from the literature.** These data are collected from free-swimming fish in their natural habitats as indicated in the method segment of the table. A total of 24 species are included. Schooling speeds given are peak and average speeds when such information is available in the original research paper. Seasonal migratory speeds are also summarized. The methods of recording speed and the associated literature reference are provided in the table. | | | | | |
| --- | --- | --- | --- | --- | --- |
| **References** | **Method** | **Species** | **School peak speed (BL s^-1^)** | **School average speed (BL s^-1^)** | **Migratory speed (BL s^-1^)** |
| Anras et al.,. *Canadian Journal of Fisheries and Aquatic Sciences.* **54**, 162-168 (1997) | Telemetry | European seabass (*Dicentrarchus labrax*) (data for two different tidal ponds) | 9.5 | 4.8 | n/a |
|  |  |  | 16.7 | 8.3 | n/a |
| Brehmer et al., *Estuaries and coasts*. **34**, 739-744.  (2011) | Sonar | Eel (*Anguilla anguilla*), big-scale sand smelt (*Atherina boyeri*), European seabass (*Dicentrarchus labrax*), gilthead sea bream (*Sparus aurata*), various mullet species *(Liza ramada)*, Mugil cephalus (*Chelon labrosus*) | 26 | n/a | n/a |
| Misund et al., *ICES Journal of Marine Science*. **49**, 325-334. (1992) | Trawling | Herring (*Clupea harengus*) | 18.6 | 13.7 | n/a |
|  |  | Herring (*Clupea harengus*) | 7.7 | 3.4 | n/a |
|  |  | Sprat (*Sprattus sprattus*) | n/a | n/a | n/a |
|  |  | Sprat (*Sprattus sprattus*) | n/a | n/a | n/a |
| Takahashi et al., *The Journal of the Marine Acoustics Society of Japan*. **43**, 145-160 (2016) | Trawling | Capelin (*Mallotus villosus*) | n/a | 4.00 | n/a |
|  |  | Capelin (*Mallotus villosus*) | n/a | 10.00 | n/a |
|  |  | Japanese anchoby (*Engraulis japonicas*) | n/a | 10 | n/a |
| Hafsteinsson & Misund  *ICES Journal of Marine Science*. ***52***, 915-924.  (1995) | Trawling | Herring (*Clupea harengus*) | n/a | 4.3 | n/a |
|  |  | Capelin (*Mallotus villosus*) | n/a | 6 | n/a |
| Misund et al.,  *African Journal of Marine Science*. **25**, 185-193. (2003) | Trawling | Sardine (Sardinops Sagax) | n/a | 5.9 | n/a |
| Gleiss et al., *Royal Society open science*. **6**, 190203.  (2019) | Motion-sensitive tags and video cameras | Atlantic bluefin tuna (*Thunnus thynnus*) | n/a | n/a | 0.60 |
| Block et al., *Journal of Experimental Biology*. **166**, 267-284 (1992) | Speedometer | Blue marlin (*Makaira nigricans*) | n/a | n/a | 0.70 |
| Carey & Scharold  *Marine biology*. **106**, 329-342  (1990) | Speedometer | Blue shark (*Prionace glauca*) | n/a | n/a | 0.8 |
| Magnuson *Fish physiology*, 240-313. (1978) | Aerial photographs - acoustic tracking | Scombroid fishes | n/a | n/a | 0.95 |
| Weihs *Copeia*. **1981**, 219-222. (1981) | Cinematography | *Carcharhinid* sharks | n/a | n/a | 0.31 |
| Marras et al., *Integrative and comparative biology*. **55**, 719-727 (2015) | High- frequency accelerometry and high-speed video observations | Sailfish (*Istiophorus albicans*) | n/a | n/a | 0.96 |
| Klimley et al.,*Environmental biology of fishes.* **63**, 117-135 (2002) | Ultrasonic transmitter tag | Marko shark (*Isurus paucus*) | n/a | n/a | 0.70 |
|  |  | White shark (*Carcharodon carcharias*) | n/a | n/a | 0.50 |
|  |  | Blue sharks (*Prionace glauca*) | n/a | n/a | 0.40 |
| Lowe *Journal of Experimental Marine Biology and Ecology.* **278**, 141-156 (2002) | Acoustic transmitter | Hammerhead shark (*Sphyrna Rafinesque*) | n/a | n/a | 0.82 |
| Sepulveda et al., *Marine Biology*. **145**, 191-199.  (2014) | Acoustic transmitter | Marko shark (*Isurus paucus*) | n/a | n/a | 0.55 |
| Fernö et al., *Marine Biology Research.* **7**, 310-313  (2011) | Tracking | Atlantic cod (*Gadus morhua*) | n/a | n/a | 0.73 |
| Tanka et al., *Journal of Experimental Biology*. **204**, 3895-3904. (2001) | Speedometer | Chum salmon (*Oncorhynchus keta*) | n/a | n/a | 1.15 |
| Kawabe et al., *ICES Journal of Marine Science*. **61**, 1080-1087. (2004) | Speedometer | Japanese flounder (*Paralichthys olivaceus*) | n/a | n/a | 0.60 |
| Watanabe & Sato  *PLoS One*. **3**, e3446.  (2008) | Speedometer | Ocean sunfish (*Mola mola*) | n/a | n/a | 0.52 |

**Supplement file 1d. Fish biometry summary table.** The wet weight of each school and each solitary individual is listed. The fork length of solitary individuals and the individuals within the school is measured. The individual length was measured using the Photron FASTCAM Viewer, v.4 software from captured images. The missing values of fork length for a few individual(s) within a school occurs because some individuals were visually blocked by others in the group.

|  | Wet weight (g) | | Individual in the school | Fork length (mm) |
| --- | --- | --- | --- | --- |
| School 1 | 13.71 | 1 | | 53.3 |
|  |  | 2 | | 47.1 |
|  |  | 3 | | 61.3 |
|  |  | 4 | | 48.8 |
|  |  | 5 | | 51.6 |
|  |  | 6 | | 50.2 |
|  |  | 7 | | 52.2 |
|  |  | 8 | |  |
| School 2 | 13.26 | 1 | | 55.3 |
|  |  | 2 | | 57.9 |
|  |  | 3 | | 56.3 |
|  |  | 4 | | 52.4 |
|  |  | 5 | | 58.3 |
|  |  | 6 | | 53.6 |
|  |  | 7 | | 55.1 |
|  |  | 8 | |  |
| School 3 | 13.51 | 1 | | 53.7 |
|  |  | 2 | | 58.6 |
|  |  | 3 | | 50.3 |
|  |  | 4 | | 58.2 |
|  |  | 5 | | 57.8 |
|  |  | 6 | | 58.5 |
|  |  | 7 | |  |
|  |  | 8 | |  |
| School 4 | 13.46 | 1 | | 59.2 |
|  |  | 2 | | 52.1 |
|  |  | 3 | | 56.4 |
|  |  | 4 | | 50.8 |
|  |  | 5 | |  |
|  |  | 6 | |  |
|  |  | 7 | |  |
|  |  | 8 | |  |
| School 5 | 13.46 | 1 | | 62.2 |
|  |  | 2 | | 58.8 |
|  |  | 3 | | 60.9 |
|  |  | 4 | | 62.3 |
|  |  | 5 | | 56.0 |
|  |  | 6 | | 65.2 |
|  |  | 7 | | 49.1 |
|  |  | 8 | |  |
| Individual 1 | 4.53 |  | | 82.8 |
| Individual 2 | 4.10 |  | | 74.4 |
| Individual 3 | 3.83 |  | | 76.7 |
| Individual 4 | 4.62 |  | | 88.0 |
| Individual 5 | 3.51 |  | | 77.7 |
